# Real-world discrimination and incremental value of first-trimester placental growth factor for hypertensive disorders of pregnancy: a retrospective Chinese cohort

**DOI:** 10.3389/fendo.2026.1960406

**Published:** 2026-10-15

**Authors:** Lingling Jiang, Minxia Chai, Yuan Xia

**Affiliations:** Hangzhou Fuyang Women And Children Hospital, Hangzhou, Zhejiang, China

**Keywords:** China, first-trimester prediction, gestational hypertension, hypertensive disorders of pregnancy, placental growth factor, preeclampsia

## Abstract

**Background:**

Placental growth factor (PlGF) contributes to first-trimester screening algorithms for preeclampsia, but its prognostic performance as an isolated biomarker in routine Chinese care is uncertain.

**Methods:**

We performed a retrospective, single-centre cohort study among pregnancy episodes recorded in a hospital PlGF workbook. Pregnancy episodes were deduplicated, PlGF values were traced to source laboratory cells, and hypertensive disorders of pregnancy (HDP) were ascertained using unvalidated free-text discharge-diagnosis keywords. The primary operational window was 9 + 0 to 13 + 6 weeks; 11 + 0 to 13 + 6 weeks was evaluated separately. We compared PlGF with a parsimonious maternal-characteristics model containing age, pre-pregnancy body mass index (BMI), and nulliparity.

**Results:**

From 405 source rows, 380 pregnancy episodes were resolved; 310 met the primary criteria, including 27 HDP events (11 cases of preeclampsia and 16 of gestational hypertension). PlGF alone had an area under the curve (AUC) of 0.633 (95% bootstrap CI 0.523–0.738), and internally derived PlGF multiple of the median (MoM) had an AUC of 0.636 (0.533–0.740). In 308 complete records, the maternal-characteristics model had an apparent AUC of 0.740 (0.635–0.837), while adding log10(PlGF) yielded an AUC of 0.748 (0.638–0.849); paired ΔAUC was 0.009 (95% CI −0.019 to 0.037).

**Conclusion:**

The findings are consistent with a modest association between lower first-trimester PlGF and subsequent HDP, but they do not support isolated clinical use or a deployable prediction model.

## 1 Introduction

Hypertensive disorders of pregnancy (HDP), particularly preeclampsia, remain major causes of maternal and perinatal morbidity. Contemporary international guidance defines preeclampsia as new-onset hypertension after 20 weeks accompanied by proteinuria, maternal organ dysfunction, or uteroplacental dysfunction (1). Earlier risk identification is clinically relevant because effective prevention depends on initiating aspirin before placental disease is clinically established (2).

Placental growth factor (PlGF) is a proangiogenic member of the vascular endothelial growth factor family. In the prevailing placental model, impaired trophoblast invasion and spiral-artery remodelling promote placental stress and excess antiangiogenic signalling; soluble fms-like tyrosine kinase-1 binds PlGF and contributes to the maternal endothelial syndrome (3–5). Reduced maternal PlGF may therefore precede clinically apparent disease, but its distribution changes rapidly with gestational age and varies by population and assay (6).

The strongest first-trimester evidence concerns multimarker algorithms combining maternal characteristics, mean arterial pressure, uterine-artery Doppler indices, and biochemical markers. Such algorithms outperform risk-factor-only approaches and can identify women who benefit from aspirin prophylaxis (2, 7–11). Translation of these findings to Chinese practice is not straightforward: Asian cohorts show differences in marker distributions and screening performance, motivating population- and platform-specific calibration (7–9, 12–14). By contrast, PlGF testing later in pregnancy among women with suspected preeclampsia serves a different diagnostic purpose and should not be conflated with first-trimester risk prediction (23–25).

The clinical question addressed here is prognostic discrimination among pregnancy episodes recorded in the available hospital PlGF dataset, rather than population-wide screening of all pregnancies. We quantified verified first-trimester PlGF discrimination, evaluated internally derived gestational-age standardisation, and estimated incremental value beyond maternal age, pre-pregnancy BMI, and nulliparity. These three variables were selected pragmatically because they were available and sufficiently complete; we therefore refer to this comparator as a parsimonious maternal-characteristics model rather than a comprehensive clinical model.

## 2 Materials and methods

### 2.1 Study design, setting, and ethics

This retrospective cohort study used routine-care records from Hangzhou Fuyang Women And Children Hospital, Zhejiang, China. The source workbook contained records of hospital pregnancy episodes with PlGF testing; it was not possible to establish whether testing was universal or selectively ordered or what proportion of all hospital pregnancies or deliveries the cohort represented. Reporting follows STROBE and, for the exploratory prediction analyses, relevant TRIPOD principles (17, 18). The Institutional Review Board approved the study (2026-K06; 21 May 2026) and waived individual consent because the analysis was retrospective. The analysis files were de-identified before statistical analysis.

### 2.2 Source audit and cohort construction

The protected source workbook contained basic information, longitudinal laboratory, and delivery sheets. Basic-information rows were treated as candidate pregnancy episodes and linked using maternal birth date, admission and discharge dates, stated gestational age, and the temporal relation between last menstrual period (LMP), sampling, and delivery. Duplicate components were consolidated into one hospital episode. Six structurally invalid rows were excluded, and five date errors were corrected only when another source sheet together with pregnancy timing uniquely supported the correction; decisions were retained in an audit ledger. The deduplicated cohort contained one pregnancy episode per woman; the 380 resolved pregnancy episodes therefore represented 380 unique women.

Eligible primary-cohort episodes required a traceable PlGF value and collection date, a recorded LMP that permitted confirmation of sampling at 9 + 0 to 13 + 6 weeks (9.0 ≤ gestational age < 14.0), a discharge outcome, and no recorded chronic hypertension or pregestational diabetes. Menstrual-cycle regularity was not recorded in the available source records. Gestational age at sampling was calculated from the documented LMP; no ultrasound, crown–rump length measurement, or other independent gestational-age assessment was available to corroborate LMP-based dating. This dependence on LMP-based dating is a limitation of the retrospective dataset. The 9-week lower bound was retained as the operational first-trimester window used in the source audit to avoid excluding traceable early tests after inspection of the results; because much of the cited screening literature focuses on 11 + 0 to 13 + 6 weeks, that narrower window was evaluated separately. The exclusions defined a target population without recorded pre-existing hypertension or diabetes, but no dated prospective protocol was available to establish that these criteria had been prespecified prospectively. A sensitivity analysis included the two chronic-hypertension episodes; no pregestational-diabetes episode was identified by the recorded diagnosis rule.

### 2.3 PlGF measurement and gestational-age standardisation

Maternal venous PlGF was measured in the hospital laboratory using the Shinei1910 automated chemiluminescence platform (Ningbo Aucheer Biotechnology Co., Ltd., China) and reported in pg/mL. Values below the reporting threshold were retained at the reported threshold and flagged; three such results occurred. Gestational age at sampling was calculated exclusively from the recorded LMP, and no ultrasound redating was applied in the analytic pipeline. Among non-HDP controls, a linear model of log10(PlGF) on gestational age generated expected PlGF values; observed values were divided by the expected values to obtain MoM. The equation was derived internally from the same cohort and was refitted within each bootstrap resample used to assess MoM AUC. The fitted equation is provided in **Supplementary Methods**.

### 2.4 Outcomes and predictors

The primary outcome was confirmed HDP, defined from free-text discharge diagnoses containing source-language terms corresponding to preeclampsia or gestational hypertension, including a common local abbreviation for gestational hypertension (15, 16). A term accompanied only by an uncertainty marker was not considered confirmed, and preeclampsia took precedence when both terms occurred (**Supplementary Table S6**). International Classification of Diseases (ICD) codes were not used, and the keyword algorithm was not validated against a chart-review subset. In this manuscript, the term “clinical” refers only to a parsimonious maternal-characteristics model comprising maternal age at PlGF sampling, pre-pregnancy BMI, and nulliparity. These variables were abstracted from routine hospital records: maternal age was calculated from the recorded date of birth and PlGF sampling date, pre-pregnancy BMI was derived from the pre-pregnancy weight and height fields, and nulliparity was abstracted from the obstetric-history field. Pre-pregnancy weight was abstracted from the clinical-record field, but information on whether it was measured or self-reported was unavailable. This model is not an established clinical risk score and does not include blood pressure, uterine-artery Doppler, or other clinical measurements. Aspirin and low-molecular-weight heparin (LMWH) exposure variables were used only in an exploratory subgroup analysis because medication fields were incomplete.

### 2.5 Statistical analysis

Continuous variables are reported as medians (interquartile range), and categorical variables as n/N (%) using available-case denominators. Normality was assessed with Shapiro–Wilk tests; in the overall primary cohort, all continuous variables reported in **Table 1** departed from a normal distribution (all P < 0.001). Descriptive group comparisons therefore used Mann–Whitney U or Fisher exact tests, but individual between-group P values are not emphasised. Because lower PlGF was associated with higher risk, discrimination used −PlGF or −PlGF MoM. Area under the curve (AUC) 95% confidence intervals were obtained with 1,000 stratified bootstrap samples.

**Table 1:** Characteristics of the verified 9 + 0 to 13 + 6-week cohort by confirmed HDP status.

Characteristic	No HDP (n = 283)	HDP (n = 27)	Overall (n = 310)	Shapiro–Wilk P (overall)
Maternal age at PlGF sampling, years	29.6 (27.4–32.7)	30.8 (29.0–34.1)	29.6 (27.5–32.9)	< 0.001
Pre-pregnancy BMI, kg/m²	21.8 (20.0–24.6)	24.8 (23.1–28.6)	22.1 (20.3–24.8)	< 0.001
Gestational age at PlGF sampling, weeks	12.4 (12.0–12.7)	12.4 (12.1–13.1)	12.4 (12.0–12.8)	< 0.001
PlGF, pg/mL	48.3 (33.9–65.0)	38.1 (23.6–52.0)	47.8 (33.3–64.5)	< 0.001
PlGF MoM	1.1 (0.7–1.4)	0.8 (0.5–1.1)	1.0 (0.7–1.4)	< 0.001
Nulliparous, n/N (%)	175/282 (62.1%)	15/27 (55.6%)	190/309 (61.5%)	—
Twin pregnancy, n/N (%)	1/283 (0.4%)	0/27 (0.0%)	1/310 (0.3%)	—
Aspirin use during pregnancy, n/N (%)	13/250 (5.2%)	5/24 (20.8%)	18/274 (6.6%)	—
LMWH use during pregnancy, n/N (%)	13/260 (5.0%)	3/25 (12.0%)	16/285 (5.6%)	—
Left-censored PlGF result, n/N (%)	2/283 (0.7%)	1/27 (3.7%)	3/310 (1.0%)	—

Values are median (IQR) or n/N (%). Shapiro–Wilk P values are for the overall cohort. Available-case denominators are shown for categorical variables; aspirin and LMWH records were incomplete. BMI, body mass index; HDP, hypertensive disorders of pregnancy; LMWH, low-molecular-weight heparin; MoM, multiple of the median; PlGF, placental growth factor.

Unpenalised logistic maximum-likelihood models compared the parsimonious maternal-characteristics model with the same model plus log10(PlGF). We reported apparent AUC, Brier score, calibration intercept, calibration slope, and bootstrap optimism-corrected AUC, intercept, and slope from 1,000 completed resamples. Incremental discrimination was summarised by paired ΔAUC with a 1,000-sample paired bootstrap confidence interval; the P value was omitted because the confidence interval included zero and the P value would have added little information. The five-group calibration plot was descriptive and highly imprecise with only 27 events. Ridge-penalised logistic regression with five-fold cross-validation was added as an exploratory stability analysis. Complete records were used; variable-specific missingness is reported in **Supplementary Table S5**. Given the sample size, event fraction, anticipated model performance, and number of parameters, all coefficient and calibration estimates are exploratory, and no model is proposed for clinical implementation (19, 20).

Exploratory analyses evaluated the 11 + 0 to 13 + 6-week window, inclusion of the two chronic-hypertension episodes, singleton pregnancies, documented absence of both aspirin and LMWH, and classification of the uncertain gestational-hypertension label as an event. The medication analysis is described as an exploratory subgroup analysis because requiring explicitly recorded non-use selected a subgroup from incomplete medication fields. Analyses were deterministic (seed 20260801) and implemented in Python 3.12 using NumPy, pandas, SciPy, scikit-learn, and Matplotlib.

## 3 Results

### 3.1 Cohort construction

The basic-information sheet contained 405 candidate rows. Six structurally invalid rows were removed, and 19 duplicate rows were collapsed, leaving 380 pregnancy episodes from 380 unique women. A traceable PlGF value was available for 353 women. Of these, 312 had verified sampling at 9 + 0 to 13 + 6 weeks; two women with chronic hypertension were excluded, yielding the primary cohort of 310 unique women, each contributing one pregnancy episode. No case of pregestational diabetes was identified by the recorded diagnosis rule. Two further records lacked BMI or nulliparity, leaving 308 women with complete records for modelling (**Figure 1**). The cohort contained 27 HDP events: 11 cases of preeclampsia and 16 of gestational hypertension.

**Figure 1:**
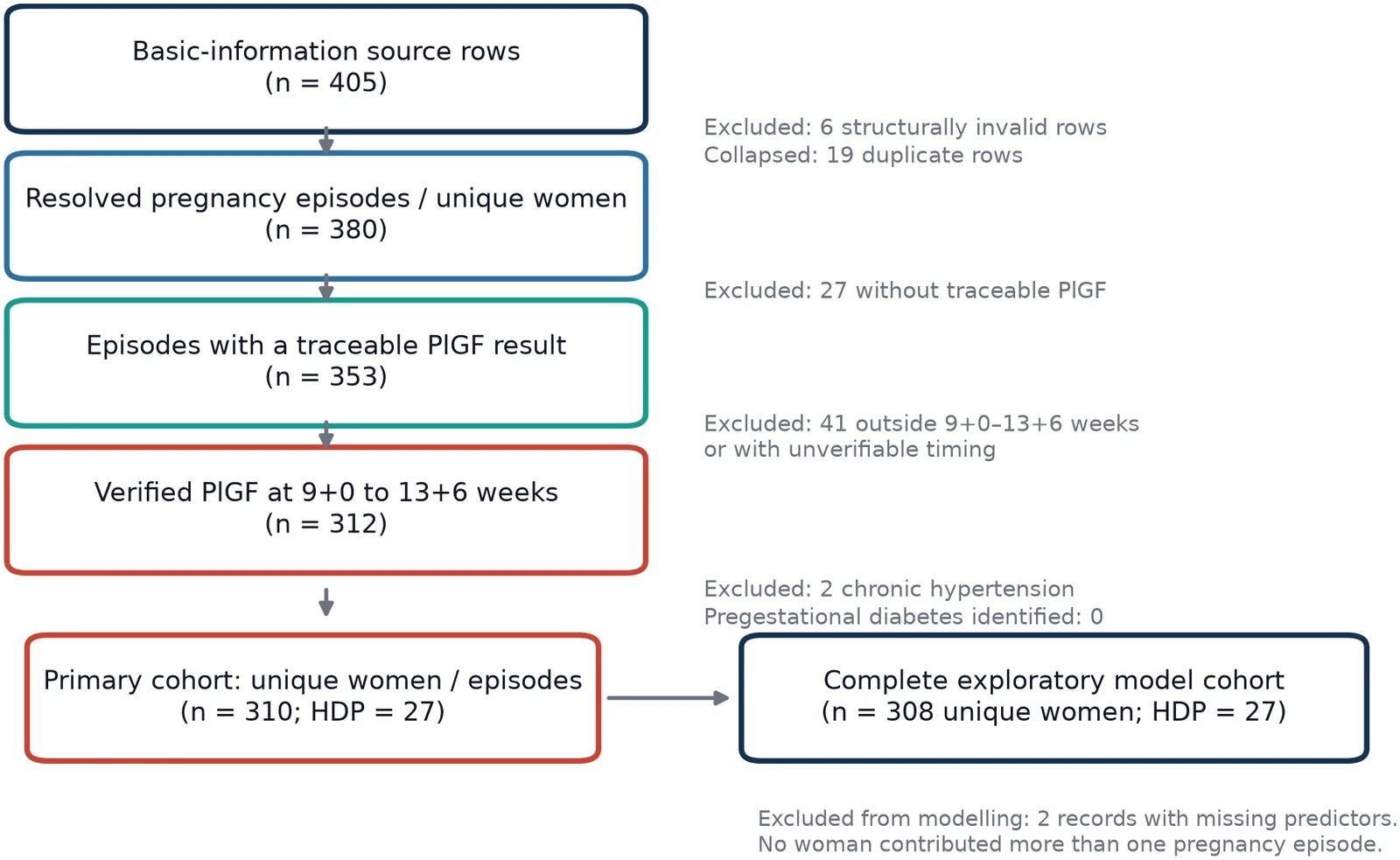
Cohort construction after source-level integrity review. Exclusions are shown adjacent to the corresponding stage. Each woman contributed one pregnancy episode; unique-women and pregnancy-episode counts were therefore identical.

### 3.2 Cohort characteristics and PlGF discrimination

Median gestational age at PlGF sampling was 12.4 weeks in both outcome groups (**Table 1**). Median PlGF was 48.3 pg/mL among non-cases and 38.1 pg/mL among women with HDP. By subtype, median PlGF was 38.1 pg/mL among women with preeclampsia and 41.7 pg/mL among those with gestational hypertension; corresponding MoM values were 0.8 and 0.9 (**Supplementary Table S1**). Aspirin and LMWH records were incomplete, and available-case denominators are shown explicitly.

PlGF alone showed modest discrimination for confirmed HDP (AUC 0.633, 95% bootstrap CI 0.523–0.738). The internally derived MoM had a similar AUC of 0.636 (0.533–0.740), indicating little gain from gestational-age standardisation in this cohort (**Table 2**, **Figure 2**). PlGF distributions by outcome subtype and by gestational age at sampling are shown in **Figures 3A, B**.

**Table 2:** Discrimination and exploratory model performance.

Analysis	N/events	Apparent AUC (95% CI)	Brier	Optimism-corrected AUC	Corrected intercept	Corrected slope	ΔAUC vs maternal characteristics (95% CI)
PlGF alone	310/27	0.633 (0.523–0.738)	—	—	—	—	—
PlGF MoM alone	310/27	0.636 (0.533–0.740)	—	—	—	—	—
Parsimonious maternal-characteristics model	308/27	0.740 (0.635–0.837)	0.073	0.719	0.014	0.927	—
Maternal characteristics + log10(PlGF)	308/27	0.748 (0.638–0.849)	0.073	0.721	0.010	0.893	0.009 (−0.019 to 0.037)

AUC confidence intervals used 1,000 stratified bootstrap samples. Apparent AUC is the in-sample estimate; optimism-corrected AUC, calibration intercept, and calibration slope were obtained from 1,000 completed bootstrap validation resamples. The paired ΔAUC confidence interval used 1,000 paired bootstrap samples. Model estimates use 308 complete records with 27 events.

**Figure 2:**
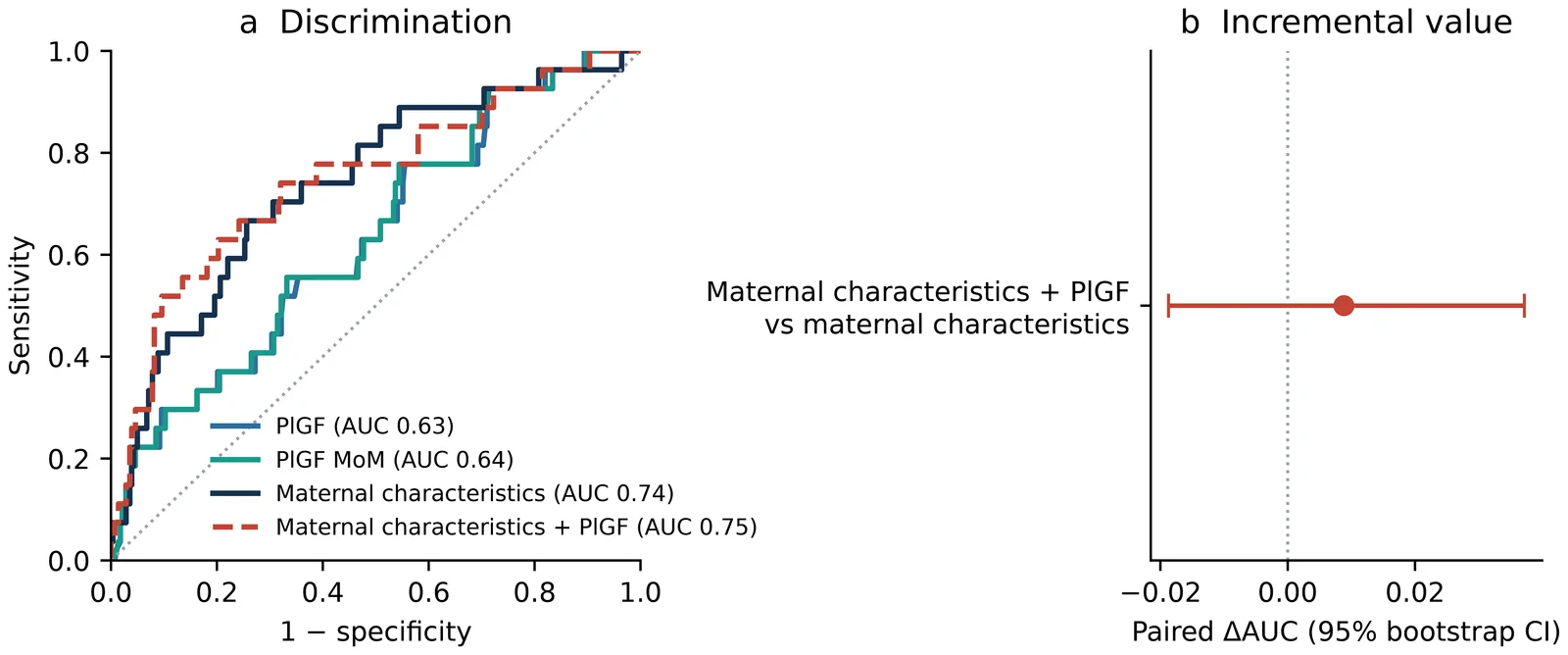
Discrimination and incremental value. **(A)** Receiver operating characteristic curves for PlGF, internally derived PlGF MoM, the parsimonious maternal-characteristics model, and the same model plus log10(PlGF). Biomarker curves use the primary cohort (n=310); model curves use complete records (n=308). **(B)** Paired ΔAUC with 95% bootstrap confidence interval.

**Figure 3:**
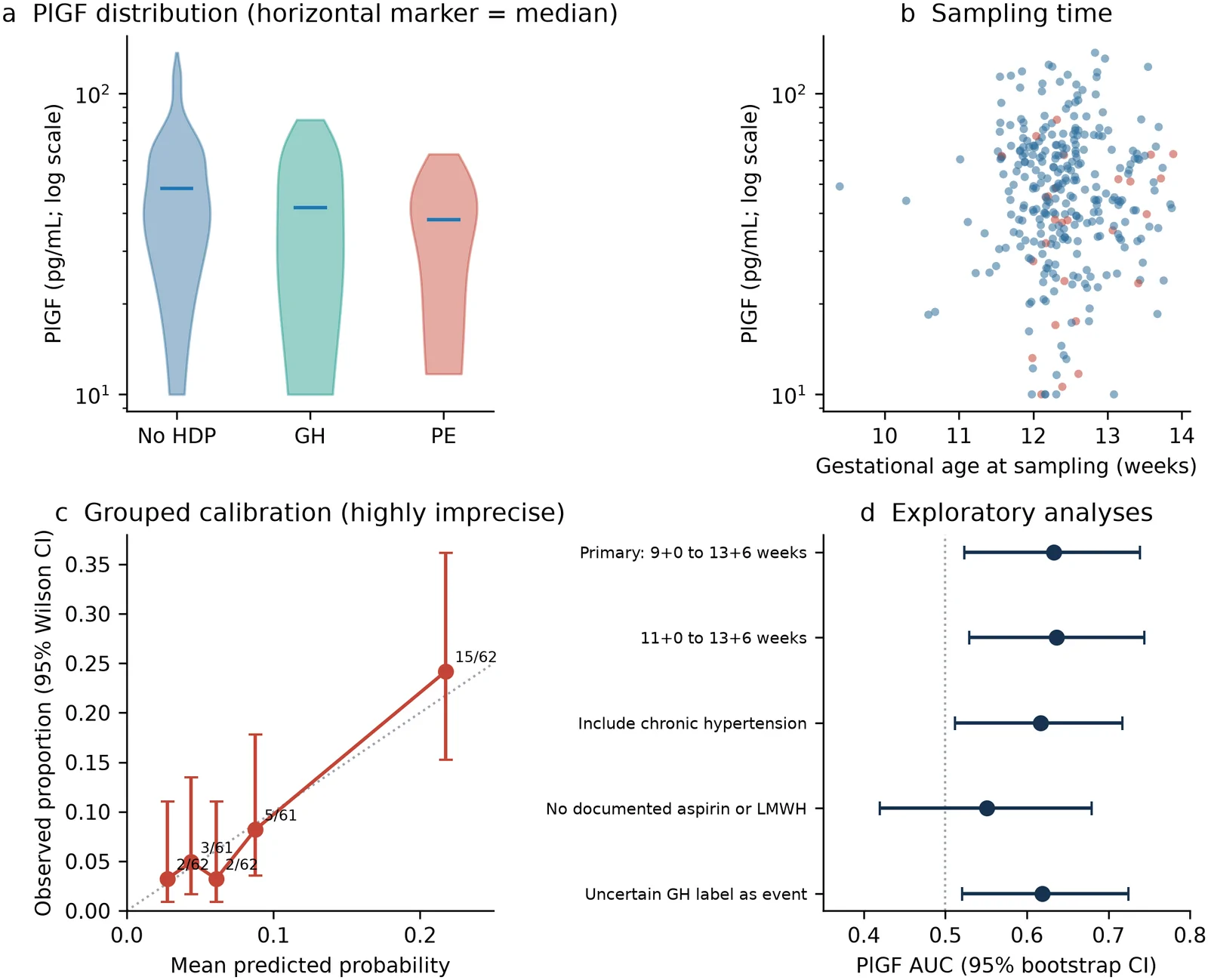
PlGF distribution, sampling time, grouped calibration, and exploratory analyses. **(A)** PlGF distribution by outcome subtype; the internal horizontal marker denotes the median. **(B)** PlGF by gestational age at sampling; red points indicate HDP. **(C)** Five-bin calibration plot for the exploratory expanded model; labels show events/total, and the plot is highly imprecise with 27 events. **(D)** PlGF AUCs across primary and exploratory analyses with 95% bootstrap confidence intervals.

### 3.3 Exploratory maternal-characteristics models

In 308 complete records with 27 events, the parsimonious maternal-characteristics model had an apparent AUC of 0.740 (95% CI 0.635–0.837), and adding log10(PlGF) yielded an apparent AUC of 0.748 (0.638–0.849). The paired ΔAUC was 0.009 (95% CI −0.019 to 0.037). Optimism-corrected AUCs were 0.719 and 0.721; corrected calibration intercepts were 0.014 and 0.010, and corrected slopes were 0.927 and 0.893 (**Table 2**; **Figure 3C**).

Among the included predictors, pre-pregnancy BMI had the largest coefficient magnitude in the full exploratory model (OR 1.17 per kg/m², 95% CI 1.06–1.28) (**Supplementary Table S2**). Maternal age, nulliparity, and log10(PlGF) had wide confidence intervals. Ridge regression produced apparent AUCs of 0.740 and 0.746 for the maternal-characteristics and expanded models, respectively, with a paired ΔAUC of 0.006 (95% CI −0.023 to 0.035; **Supplementary Tables S3, S4**). These coefficients are reported for transparency and should not be interpreted as stable clinical effect estimates.

### 3.4 Sensitivity analyses

The PlGF AUC was 0.636 in the 11 + 0 to 13 + 6-week analysis and 0.617 when the two chronic-hypertension episodes were included. The subgroup with documented non-use of aspirin and LMWH contained 242 episodes and 17 events, with an AUC of 0.551; incomplete medication fields and subgroup selection preclude interpretation of this analysis as a conventional robustness analysis. Treating the uncertain gestational-hypertension label as an event yielded an AUC of 0.619. The singleton-pregnancy result is reported in **Supplementary Table S7** because only one twin pregnancy was present (**Table 3**; **Figure 3D**).

**Table 3:** Exploratory analyses of PlGF stand-alone discrimination.

Scenario	N	HDP events	PlGF AUC	95% CI
Primary analysis (9 + 0 to 13 + 6 weeks)	310	27	0.633	0.523–0.738
11 + 0 to 13 + 6 weeks	306	27	0.636	0.529–0.744
Include chronic-hypertension episodes	312	29	0.617	0.512–0.717
Documented no aspirin or LMWH exposure	242	17	0.551	0.419–0.679
Treat uncertain GH label as an event	310	28	0.619	0.521–0.725

The documented no-exposure subgroup required both aspirin and LMWH fields to be zero; records with missing medication status were not assumed unexposed. The singleton result is reported in **Supplementary Table S7**. GH, gestational hypertension.

## 4 Discussion

### 4.1 Principal findings

After reconstructing pregnancy episodes and verifying PlGF timing, lower first-trimester PlGF was associated with subsequent HDP and provided modest stand-alone discrimination (AUC approximately 0.63). Internal gestational-age standardisation did not materially change performance. The parsimonious maternal-characteristics model showed better discrimination than PlGF alone, while adding PlGF increased AUC by less than 0.01 with a confidence interval that included zero. BMI showed the strongest association among the included predictors, but their relative contributions to discrimination were not formally decomposed. The findings are consistent with a modest association between lower first-trimester PlGF and subsequent HDP, but do not indicate accurate stand-alone classification or support a deployable prediction model.

### 4.2 Interpretation in context

These findings are compatible with the architecture of established first-trimester screening, in which PlGF is combined with maternal history, mean arterial pressure, uterine-artery Doppler, and calibrated biomarker distributions (2, 7–11). The composite endpoint may have attenuated or obscured outcome-specific associations because preeclampsia and gestational hypertension are related but biologically distinct. Descriptive subtype values are therefore reported, but the small event counts precluded separate prediction modelling.

The association with BMI is clinically plausible and is consistent with epidemiological evidence linking increasing pre-pregnancy adiposity to preeclampsia risk (21, 22). The maternal-characteristics model discriminated better than PlGF alone, and BMI showed the strongest association among the included predictors; however, the analysis did not formally decompose each predictor’s contribution to AUC. The coefficients should not be interpreted causally.

Later-pregnancy PlGF or sFlt-1/PlGF testing among symptomatic women serves short-term diagnostic triage (23–25) and should not be conflated with first-trimester prognostic discrimination. Because the mechanism by which women entered the PlGF workbook and the hospital-wide denominator were unavailable, this cohort cannot be described as an unselected population.

### 4.3 Strengths and limitations

A principal strength is the explicit data-integrity audit. We did not treat 405 source rows as 405 independent women: duplicate hospital episodes were resolved, malformed rows were excluded, laboratory blocks were matched one-to-one on the basis of pregnancy timing, and all corrections were documented. The analyses can be reproduced using the de-identified cohort and deterministic script. We also distinguished the biomarker cohort from the complete exploratory-model cohort and reported the actual number of completed bootstrap iterations.

Several limitations are important. This was a retrospective single-centre study assembled from available PlGF testing records, with uncertain selection into testing and no hospital-wide denominator. Outcomes were extracted from unvalidated free-text discharge diagnoses rather than independently adjudicated criteria; ICD codes and a validation chart-review subset were unavailable. Combining preeclampsia and gestational hypertension may have obscured subtype-specific associations. MoM was internally derived, gestational age was based on the recorded LMP without ultrasound redating, and whether pre-pregnancy weight was measured or self-reported was unknown. Medication fields were incomplete, and the small number of events (n = 27) makes coefficients, calibration, and subgroup estimates unstable despite internal resampling. External validation was not performed.

### 4.4 Implications

An isolated PlGF threshold from this cohort should not be used for routine risk classification. Prospective work should document the testing pathway, prespecify outcome adjudication, collect mean arterial pressure measurements and uterine-artery Doppler indices, standardise the assay, record medication exposure completely, and recruit a sufficient number of events to meet calibration-focused sample-size targets.

## 5 Conclusion

The findings are consistent with a modest association between lower first-trimester PlGF and subsequent HDP among pregnancy episodes recorded in the available hospital PlGF testing dataset. PlGF showed modest stand-alone discrimination and added little to a parsimonious maternal-characteristics model. These results support prospective evaluation within calibrated multimarker pathways rather than isolated clinical use.

## Data Availability

The raw data supporting the conclusions of this article will be made available by the authors, without undue reservation.
