# Supplementary material for: Real-world discrimination and incremental value of first-trimester placental growth factor for hypertensive disorders of pregnancy: a retrospective Chinese cohort
Source: Front Endocrinol (Lausanne). 2026 Oct 15;17:1960406. doi: 10.3389/fendo.2026.1960406 (PMC13716667; doi:10.3389/fendo.2026.1960406)
Supplement: Supplementary file 1 [file Table1.docx]

**Supplementary Material**

Manuscript 1960406

# Supplementary Methods

Internally derived PlGF MoM equation: log10(expected PlGF) = 1.5786235296 + 0.0068074022 × gestational age in weeks. The equation was estimated among 283 non-HDP controls. Within the MoM AUC bootstrap, this relationship was refitted in each resample before calculating expected PlGF and MoM.

# Supplementary Table S1. PlGF by outcome subtype

| **Outcome group** | **N** | **PlGF, pg/mL** | **PlGF MoM** |
| --- | --- | --- | --- |
| No HDP | 283 | 48.3 (33.9–65.0) | 1.1 (0.7–1.4) |
| Gestational hypertension | 16 | 41.7 (23.7–62.1) | 0.9 (0.5–1.3) |
| Preeclampsia | 11 | 38.1 (24.6–48.5) | 0.8 (0.5–1.0) |

# Supplementary Table S2. Unpenalised logistic-regression coefficients

These exploratory estimates are reported for transparency and are not proposed for clinical use.

| **Model** | **Predictor** | **OR (95% CI)** |
| --- | --- | --- |
| Clinical | Maternal age (per year) | 1.089 (0.981–1.209) |
| Clinical | Pre-pregnancy BMI (per kg/m²) | 1.194 (1.092–1.306) |
| Clinical | Nulliparity | 1.313 (0.491–3.510) |
| Clinical + PlGF | Maternal age (per year) | 1.090 (0.981–1.210) |
| Clinical + PlGF | Pre-pregnancy BMI (per kg/m²) | 1.165 (1.060–1.280) |
| Clinical + PlGF | Nulliparity | 1.374 (0.505–3.737) |
| Clinical + PlGF | log10(PlGF) | 0.305 (0.054–1.729) |

# Supplementary Table S3. Ridge-regression performance

| **Analysis** | **N/events** | **Apparent AUC** | **95% CI lower** | **95% CI upper** | **Brier score** | **Selected C** | **Paired delta AUC** | **Paired delta 95% CI lower** | **Paired delta 95% CI upper** |
| --- | --- | --- | --- | --- | --- | --- | --- | --- | --- |
| Parsimonious maternal-characteristics ridge model | 308/27 | 0.740 | 0.635 | 0.838 | 0.073 | 0.391 | — | — | — |
| Parsimonious maternal-characteristics + PlGF ridge model | 308/27 | 0.746 | 0.635 | 0.847 | 0.073 | 0.184 | 0.006 | −0.023 | 0.035 |

# Supplementary Table S4. Ridge-regression coefficients

| **Model** | **Predictor** | **Standardized beta** | **OR per SD** |
| --- | --- | --- | --- |
| Parsimonious maternal-characteristics ridge model | Maternal age (per year) | 0.311 | 1.365 |
| Parsimonious maternal-characteristics ridge model | Pre-pregnancy BMI (per kg/m²) | 0.646 | 1.909 |
| Parsimonious maternal-characteristics ridge model | Nulliparity | 0.093 | 1.097 |
| Parsimonious maternal-characteristics + PlGF ridge model | Maternal age (per year) | 0.276 | 1.318 |
| Parsimonious maternal-characteristics + PlGF ridge model | Pre-pregnancy BMI (per kg/m²) | 0.523 | 1.687 |
| Parsimonious maternal-characteristics + PlGF ridge model | Nulliparity | 0.079 | 1.082 |
| Parsimonious maternal-characteristics + PlGF ridge model | log10(PlGF) | −0.254 | 0.776 |

# Supplementary Table S5. Data completeness

| **Variable** | **Available, n** | **Missing, n** | **Missing, %** |
| --- | --- | --- | --- |
| Maternal age at PlGF sampling | 310 | 0 | 0.0 |
| Pre-pregnancy BMI | 309 | 1 | 0.3 |
| Nulliparity | 309 | 1 | 0.3 |
| PlGF | 310 | 0 | 0.0 |
| Aspirin exposure | 274 | 36 | 11.6 |
| LMWH exposure | 285 | 25 | 8.1 |

# Supplementary Table S6. Outcome keyword rules

| **Outcome** | **Free-text terms** | **Rule** |
| --- | --- | --- |
| Preeclampsia | 子痫前期 | Confirmed term without a trailing uncertainty marker |
| Gestational hypertension | 妊娠期高血压; 妊高症 | Confirmed term without preeclampsia and without a trailing uncertainty marker |

# Supplementary Table S7. Singleton exploratory analysis

| **Scenario** | **N** | **HDP events** | **PlGF AUC** | **95% CI** |
| --- | --- | --- | --- | --- |
| Singleton pregnancies | 309 | 27 | 0.632 | 0.527–0.732 |
